# Extraction, Characterization, Antioxidant, and Immunostimulatory Activities of Polysaccharides from* Hedyotis corymbosa*

**DOI:** 10.1155/2018/8617070

**Published:** 2018-11-14

**Authors:** Li-mei Lin, Su-hui Xiong, Ling-jia Zhao, Jie Tang, Zhi-min Zhang, Ya-mei Li, Tao Zheng, Bo-hou Xia

**Affiliations:** ^1^College of Pharmacy, Hunan University of Chinese Medicine, Changsha 410208, China; ^2^Collaborative Innovation Center for the Protection and Utilization of Chinese Herbal Medicine Resources in Hunan Province, Hunan University of Chinese Medicine, Changsha 410208, China

## Abstract

In this study, optimization of enzyme-assisted extraction, purification, characterization, and its bioactivities of polysaccharides from* Hedyotis corymbosa* (HCP) was investigated. It was found that the optimum extraction conditions were 3% of enzyme concentration (*X*_*1*_), 30 of liquid-to-solid ratio (*X*_*2*_), 56°C of extraction temperature (*X*_*3*_), 200W of ultrasonic power (*X*_*4*_), 10 min of extraction time* (X*_*5*_), and 5 of pH value (*X*_*6*_). Under optimum conditions, the experimental yield (4.10 ± 0.16%) was closed to the predicted value (4.02%). The crude HCP was further purified using DEAE-52 and Sephadex G-150 gel column, and a major polysaccharide fraction from HCP, designed as HCP-1a with molecular weight of 33.9 kDa, was obtained. The HCP and HCP-1a were characterized by chemical analysis, FT-IR, and HPLC. For antioxidant activities* in vitro*, HCP possessed strong hydroxyl radical scavenging, DPPH radical scavenging, and Fe^2+^ chelating activities. In subsequent immunostimulatory studies, significantly decreased NO, IL-1*β*, and TNF-*α* concentrations were observed in both of HCP and HCP-1a treated RAW264.7 cells. Therefore, this study may indicate some insights into the application of polysaccharides from* Hedyotis corymbosa* as potential natural antioxidants and immunostimulants.

## 1. Introduction

The genus* Hedyotis* (family of Rubiaceae) are mainly distributed in the tropical and subtropical region of the world [1]. In the traditional medicine, over 20* Hedyotis* species have been used for treatment of diseases and healing practices.* Hedyotis corymbosa *is one of the most popular species, which is regarded as the active ingredients in several Chinese herbal medicines [2, 3].* Hedyotis corymbosa* is taken for treating arthralgia, tumor, fever, and jaundice [4–6]. Recently, some research reports on* Hedyotis corymbosa (HC)* and its compositions expression antifungal, antioxidant, anti-injury, analgesic, and liver protection [5, 7, 8]. According to the preparation process and primary active ingredients of previously studies, the proteins, carbohydrates, phenols, iridoids, tannins, flavonoids, saponins, steroids, terpenoids, and glycosides were the main focus and accounted for the major constituents [9, 10]. However, research into some other components like polysaccharides is quite limited.

Functional components from plants, especially polysaccharides, have gained much popularity on account of their broad spectrum of biological activities and pharmacological effects, such as antitumor, antioxidant, antimicrobial, and anti-inflammatory activities [11–13]. Among these, interest is growing recently in the antitumor and immunity-stimulating activities of natural polysaccharides due to their relatively low toxicities and few side effects [14, 15]. Immunity plays a vital role in the balance of nutrition and infection. The destruction of balance between immunity, nutrition, and infection may lead to high morbidity and mortality, such as cancer or autoimmune diseases [16]. Immunostimulatory therapy has long been considered an important feature of improving the body's nonspecific defense. Therefore, natural polysaccharides were popular ingredient as medicine and health products.

This study firstly aimed to optimize the extraction conditions for polysaccharides of* Hedyotis corymbosa *(HCP) based on Plackett-Burman statistical design, paths of steepest ascent method, and central composite rotatable design. Moreover, the crude HCP was separated and purified by DEAE-52 cellulose and Sephadex G-150. All of the obtained polysaccharides were further characterized via chemical analysis (UV, HPLC, FT-IR, or HPGPC). Furthermore, the* in vitro *antioxidant activities (ABTS, FRAP, and DPPH) and immunostimulatory activities (IL-1*β*, TNF-*α*, and NO) of crude HCP and its purified fractions were also discussed.

## 2. Materials and Methods

### 2.1. Materials and Chemicals

*Hedyotis corymbosa* (HC) was bought from GaoQiao natural herbal special market (ChangSha, China). Dried sample was pulverized by a disintegrator and screened to obtain the powder sample. All samples were stored in a desiccator before used.

3-(4,5-Dimethylthiazol-2-yl)-2,5-diphenyltetrazolium bromide (MTT), Dextrans of different molecular weights, 2,2-diphenyl-1-picrylhydrazyl (DPPH), and lipopolysaccharide (LPS) were obtained from Sigma Chemical Co. (USA). Total antioxidant capacity assay kit (ABTS, FRAP) was obtained from Beyotime Institute of Biotechnology (Jiangsu, China).

### 2.2. Preparation of HCP and Determination of the Yield

In the three-neck flask, HC powder was pretreated with petroleum ether and subsequently treated with 80% ethanol for two times (60 C, 5h) in order to remove some small molecules and colored materials. Finally, under centrifuge conditions (5000 rpm / min, 10 min), the samples were separated from the mixed solvent and dried until the weight was constant. For ultrasound-assisted enzymatic treatment, the sample was put into triangular flask. The extraction was performed under optimum liquid-to-solid, enzyme concentration (cellulase), ultrasonic power, pH value, extraction time, and temperature. After the ultrasound treatment, samples were treated by centrifugation (5,000 rpm, 10 min). Under the vacuum environment, the supernatant was further concentrated in order to determinate solvents volume. The concentrate was incubated for 12 h at 4°C in a fixed concentration of 80% (v/v) by using 100% (v/v) ethanol to gain the crude HCP. Determining the extraction efficiency of HCP by phenol-sulfuric acid method [17], the HCP (%) is measured as follows:

(1) HCP%=the  polysaccharides  in  extractiongweigh  of  Cornus  officinalis  powderg×100%

### 2.3. Experimental Design

Initially, a 12-run Plackett-Burman experimental design (PBD) was applied to evaluate the tested factors, such as enzyme concentration (*X*_*1*_), liquid-to-solid ratio (*X*_*2*_), extraction temperature (*X*_*3*_), ultrasonic power (*X*_*4*_), extraction time (*X*_*5*_), and PH (*X*_*6*_). For each factor, two levels were designated: -1 for the low level and +1 for the high level. HCP yield was designed as the response value in current experiment. The design of the experiment for screening of the variables was presented in Supplementary Table 1 and the variables were mentioned and denoted as numerical factors.

In view of the above-mentioned results of the PBD, the path of steepest ascent was used to obtain the center point of the response by properly altering the value of the important variables [18]. The -1 level of PBD was designed as base point of the path. All tests ended at the steepest ascent path when the response indicated no further increase. Moreover, this point is confirmed to be the center point for central composite design (CCD).

In order to optimize the extraction parameters to get optimum conditions of HCP, a CCD and response surface methodology were further performed. Based on the results from PBD and the path of steepest ascent, CCD was applied with three variables, which have significant influence on the yield of HCP. Each variable was investigated at five levels, coded as -1.682, -1, 0, +1, and +1.682, respectively. All the tests designed for the study were indicated in Supplementary Table 2. The HCP yield was fitted in a second-order polynomial equation, and the quadratic equation is as follows:

(2) $$\begin{array}{c} Y=\varphi_{0}+\overset{n}{\underset{i=1}{\sum }}\varphi_{i}x_{i}+\overset{n}{\underset{i=1}{\sum }}\varphi_{ii}{x_{i}}^{2}+\overset{n-1}{\underset{i=1}{\sum }}\overset{n}{\underset{j=i+1}{\sum }}\varphi_{ij}x_{i}x_{j} \end{array}$$

where* Y*, *φ*_0_, *φ*_i_, *φ*_ii_, and *φ*_ij_ indicate the predicted HCP yield, the intercept, the linear coefficient, the quadratic coefficient, and the interaction coefficient, respectively. Some indexes were applied to identify the experiment-based model, such as determination coefficient (*R*^*2*^), F-value, and a lack of fit [19].

### 2.4. Fractionation and Purification of Crude HCP

The crude HCP was pretreated through centrifugal machines in order to obtain clear solutions which were appropriate for the following fractionation and purification. Firstly, the fractionation was tested using DEAE-52 cellulose column (3.0 cm × 50 cm). Further experiments were carried out by eluting with NaCl in stepwise in different concentrations (0-0.5 mol/L, 1.0 mL/min). A main fraction named HCP-1 was obtained and concentrated. This main fraction was further dissolved and then loaded to a Sephadex G-150 gel column (3.0 cm × 80 cm). The fraction was further purified by eluting with 0.1 mL/min NaCl at 0.6 mL/min. Finally, another fraction abundant in polysaccharides was obtained, dialyzed, and freeze dried, yielding HCP-1a.

### 2.5. Analysis of Polysaccharides Characterization

#### 2.5.1. Preliminary Characterization of Polysaccharides

Phenol-sulfuric acid method was applied to determine the total polysaccharide applying glucose. Bradford method was applied to determine the protein content in polysaccharides applying bovine serum albumin (BSA) as a standard [20]. Hydroxy biphenyl method was used to determine the uronic acid content in polysaccharides applying bovine D-glucuronic acid as a standard [21]. Barium chloride–gelatin method was devoted to determine the sulfuric radical content in polysaccharides applying potassium sulphate as a standard [22]. Folin-Ciocalteu method was applied to determine the total polyphenols content in polysaccharides applying bovine total polyphenols as a standard [23]. The results were expressed as mg gallic acid equivalent (GAE) per mg dry sample (mg GAE /100 mg dried extract).

#### 2.5.2. Measurement of Molecular Weight (Mw)

The Mw of HCP-1A was analyzed with TSK gel G5000 column (7.8 mm *∗* 300 mm, 5 u m) by reverse phase high performance gel permeation chromatography (7.8 mm × mm, 5 u m), eluting with 0.002 M NaH_2_PO4 solution (0.05% NaN_3_) under 0.6 mL/min. Refractive index detector was applied to detect the signal. Diverse standard Dextrans (Mw: 1000, 12000, 50000, 270000, 670000, and 1100000 Da) was used to establish calibration curve. The equation of the calibration curve was log M w = −0.442T + 10.78 (T represents retention time, R^2^ = 0.9939)

#### 2.5.3. Analysis of Monosaccharides Compositions

According to previous research, monosaccharides compositions of HCP and HCP-1a were analyzed by high performance liquid chromatography (HPLC) after precolumn derivatization [24]. Mannose (Man), galacturonic acid (GalUA), rhamnose (Rha), xylose (Xyl), glucuronic acid (GlcUA), ribose (Rib), arabinose (Ara), glucose (Glc), galactose (Gal), and fucose (Fuc) were used as reference sugars.

#### 2.5.4. FT-IR Spectrometric Analysis

An ATR-FTIR (Nicolet, USA) spectrum of the extracted polysaccharides was applied. All polysaccharides were pretreated at 40°C for 24 h under the condition of vacuum before FT-IR analysis. All spectra were obtained using 64 scans in the frequency range of 525–4000 cm^−1^.

### 2.6. Assay of Antioxidant Activity* In Vitro*

The FRAP and ABTS+ radical scavenging activity of HCP and HCP-1a solutions were determined using a commercially available assay kit on the basis of the manufacturer's instructions. The FRAP results were expressed as FeSO_4_ equivalent per mg of dry weight (mM FeSO_4_/mg dried extract). The above experiment was repeated three times. DPPH radical scavenging activity was measured as described by a previous reported method [25].

### 2.7. Assay of Anti-Inflammatory Effect* In Vitro*

#### 2.7.1. Cell Treatment and Viability Analysis

RAW264.7 cell was obtained from Central South University (Changsha, China). Cells were cultured with Dulbecco's modified Eagle medium (DMEM), supplemented with 10% fetal bovine serum in flasks and cultured at 37°C in a humidified incubator containing 5% CO2. To reduce the infection of RAW264.7, 100 U/mL penicillin and 100 *μ*g/mL streptomycin were added. The effects of HCP and HCP-1a were determined by MTT assay through a previously reported method [26].

#### 2.7.2. Determination of TNF-*α*, IL-1*β* and NO

The cells were seeded into 96-well plates at 5 × 10^4^ cells/well and cultured at 37°C with 5% CO_2_. After seeding for 24 h, 100 *μ*L DMEM medium with different concentrations of HCP and HCP-1a (0, 5, 10, 20, 40, and 80 *μ*g/mL) was added to different wells. And they were subsequently incubated for another 24 h. LPS (1 *μ*g/mL) was then added and incubated for another 24 h. Finally, the culture medium was collected for further analysis. The TNF-*α*, IL-1*β*, and NO were quantified by ELISA kits (Becton Dickinson Medical Devices Co. Ltd., China) according to the instruction of manufacturers.

### 2.8. Statistical Analysis

All data were expressed as $\overset{-}{X}$  ± SD from triple-repeat experiments. The significance difference was tested using Student's t-test and one-way analysis of variance (ANOVA).* p* < 0.05 and* p* < 0.01 were considered statistically significant differences.

## 3. Result and Discussion

### 3.1. Optimization of HCP Production by Plackett-Burman Design (PBD)

The importance of the six parameters for HCP yield was studied by PBD. Factors with p<0.05 were considered to be significant parameters on the response and therefore were included in the further optimization tests. As shown in Supplementary Table 1, the results showed that the extraction temperature, enzyme concentration, and liquid-to-solid ratio had significantly effect on HCP yield (p < 0.05). Moreover,* R*^*2*^ was found to be 90.56%, which indicated low amount of total variation in the response results unexplained by the model. In order to improve economic efficiency, extraction time and ultrasonic power were selected to be 10 min and 200W, respectively. In addition, the PH was fixed at 5.0 because by this value the reaction conditions were relatively mild.

### 3.2. Screening by Path of Steepest Ascent Method

For each of the three significant factors, the values of low level from PBD tests were applied as initial points. The path of steepest ascent started from this point and moved along with the path through increasing or decreasing the values of the three significant factors. As shown in Supplementary Table 3, the maximum production of HCP was obtained when the parameters were enzyme concentration 3%, extraction temperature 50°C, and liquid-to-solid ratio 30 mL/g. Hence, these conditions were set as the center point of the response surface method, in view of which the response surface optimization tests were designed.

### 3.3. Optimization by Central Composite Design (CCD)

The respective design matrix and the corresponding experimental data were shown in Supplementary Table 2. The main effects and the interactions between factors were assessed following ANOVA through Design Expert software 8.0.6 (Table 1). The result indicated that the three significant factors (*P *< 0.0001) explained 99.44% of the model variability, showing a high degree of correlation between the observed and predicted responses. The large adjusted* R*^*2*^ (98.94%) also validate a good relationship between the experimental data and the fitted model. Moreover, the value of lack of fit (p=0.4574) implied that the model adequately was fitted to the significant independent variable effects. The quadratic polynomial regression equation referring to the three independent variables (coded) is as follows:

(3) Y=3.98+0.14×x1+0.048×x2+0.16×x3−0.42×x1×x2−0.25×x1×x3−0.079×x2×x3−0.43×x12−0.57×x22−0.14×x32

**Figure 1:**
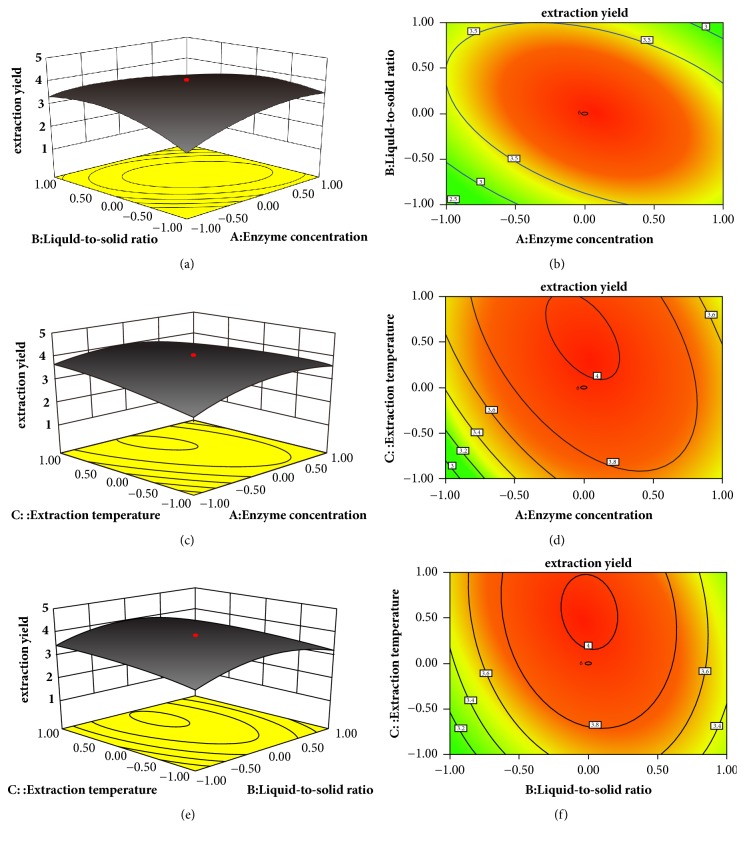
3D Response surface plots (a, c, e) and 2D contour plots (b, d, f) (*X*_*1*_: enzyme concentration, %;* X*_*2*_: liquid-to-solid ratio;* X*_*3*_: extraction temperature, °C).

To further discuss the mutual interactions and explain the results of statistical analyses, the 3D and 2D plots were obtained on the basis of the model equation (see (3)). As shown in Figures 1(a) and 1(b), the HCP yield increased as enzyme concentration and liquid-to-solid ratio increased to optimum values and then decreased with further increases of them. The 2D contour plot showed a clearly elliptical contour, suggesting a significant interaction between* X*_*1*_ and* X*_*2*_ on HCP yield. Moreover, the optimum values of* X*_*1*_ and* X*_*2*_ were observed inside the experimental boundary as there was an obvious peak in the 3D response surface curve. The similar results occurred between* X*_*1*_ and* X*_*3*_, which was shown in Figures 1(c) and 1(d), as well as interaction between* X*_*2*_ and* X*_*3*_, which was shown in Figures 1(e) and 1(f).

According to the results of statistically designed experiments, the optimum values were as follows: 2.99% of* X*_*1*_, 30.06 of* X*_*2*_, and 55.71°C of* X*_*3*_. At this point, HCP yield predicted by the model was 4.02%. Considering the feasibility of the practical process, the optimal conditions were amended as follows: 3%* X*_*1*_, 30 of* X*_*2*_, and 56°C of* X*_*3*_.

To confirm the validation of the optimization results, the optimized process was repeated three times to extract HCP. The result showed that the experimental HCP yield (4.10 ± 0.16%) agreed well with the predicted value.

### 3.4. Isolation and Purification of HCP

**Figure 2:**
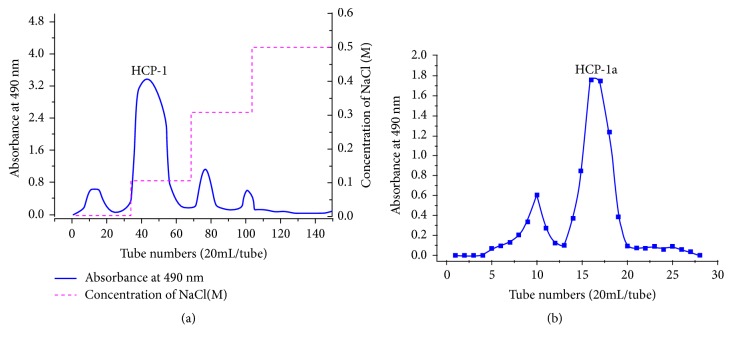
(a) Elution profile from HCP based on DEAE-52 column with water and gradient NaCl solutions (0.1, 0.3, and 0.5 M); (b) purification result of HCP-1a on Sephadex G-150 gel column with 0.1 mL/min NaCl.

The crude HCP described above was fractionated by DEAE–52 column. After an aqueous solution was applied to the column, it was eluted stepwise with NaCl solutions (0.1, 0.3, and 0.5 M). Three fractions were obtained, namely, HCP-1, HCP-2, and HCP-3 (Figure 2(a)). HCP-1 was collected for subsequent purification on account of the low yield of HCP-2 (<15mg) and HCP-3 (<10mg). The HCP-1 was then loaded onto a Sephadex G-150 gel column. HCP-1 produced two elution peaks (Figure 2(b)). The highest peak, namely, HCP-1a, was collected, dialyzed, and lyophilized.

### 3.5. Characterization of HCP and HCP-1a

#### 3.5.1. Preliminary Characterization of Polysaccharides

As shown in Table 2, the carbohydrate contents in HCP and HCP-1a were 70.36 ± 3.86% and 60.95 ± 3.05%, respectively. No protein and a small amount of total polyphenols (0.49 ± 0.02) were detected in HCP-1a, indicating that the isolation and purification of HCP by using DEAE-52 and Sephadex G-150 was efficient and useful. Moreover, HCP and HCP-1a had high content of uronic acid (21.88 ± 1.06% for crude HCP, 35.26 ± 1.51% for HCP-1a), suggesting that they were acid polysaccharide.

#### 3.5.2. Monosaccharide Composition of Crude HCP and HCP-1a

**Figure 3:**
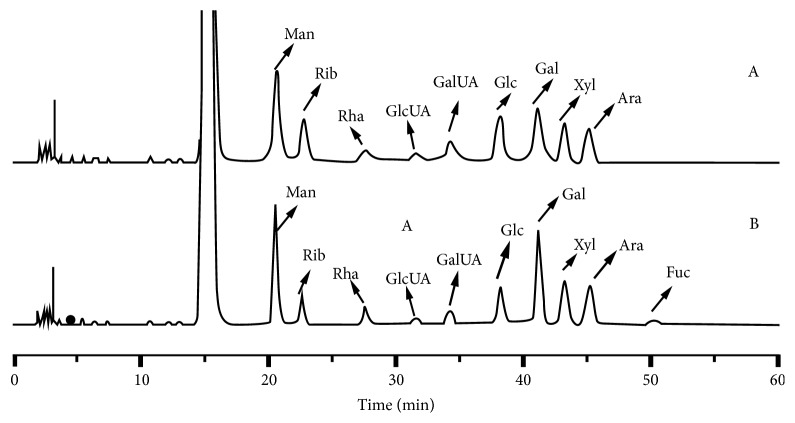
The HPLC chromatograms of component monosaccharides released from HCP (A) and HCP-1a (B) compared with standard monosaccharides (Man, mannose; Rib, ribose; Rha, rhamnose; GlcUA, glucuronic acid; GalUA, galacturonic acid; Glc, glucose; Gal, galactose; Xyl, xylose; Ara, arabinose; Fuc, fucose).

The Mw of HCP-1a was determined by HPGPC, which has been shown to be an effective method [21]. As shown in Table 2, the Mw of HCP-1a was calculated as 33.9 kDa according to the calibration curve. The typical chromatogram of crude HCP and HCP-1a sample was shown in Figure 3. Various ratios and types of monosaccharide compositions in both of polysaccharides were observed. According to the data presented in Table 2, HCP-1a had one more monosaccharide composition than that of HCP. HCP-1a consist of Man, Rib, Rha, GlcUA, GalUA, Glc, Gal, Xyl, Ara, and Fuc, with molar percentages of 21.60%, 7.85%, 5.25%, 2.79%, 3.24%, 9.79%, 23.28%, 13.88%, 9.67%, and 2.65%, respectively. Besides Fuc, HCP consist of Man, Rib, Rha, GlcUA, GalUA, Glc, Gal, Xyl and Ara in a molar percentage of 23.85, 10.52, 4.45, 3.83, 6.85, 16.94, 2.86, 15.50 and 15.20, respectively. The results indicated that HCP and HCP-1a were acidic polysaccharide.

#### 3.5.3. FT-IR Spectroscopy

**Figure 4:**
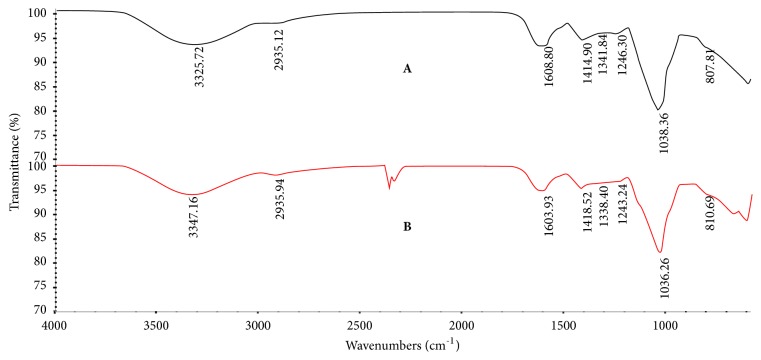
FT-IR spectrum of crude HCP (A) and HCP-1a (B).

The infrared spectra of HCP and HCP-1a ranging from 525 cm^−1^ to 4000 cm^−1^ were shown in Figure 4. Signals close to 3420, 2920, 1620, 1400, 1100, and 810 cm^−1^ were the characteristic absorption band of polysaccharides [27]. The absorption peaks of coarse HCP and HCP-1A near 1605 cm^−1^ and 1415 cm^−1^ are related to the tensile vibration of carbonyl bond ester carbonyl groups (C = O) and carboxyl groups (COO-), indicating that crude HCP and HCP-1A were acidic polysaccharides. The strong absorption band of 1035 cm^−1^ (1038 cm^−1^ for HCP and 1036 cm^−1^ for HCP-1a) was the stretching vibration of C-O-C and C-O-H caused by the sugar ring. Moreover, absorption close to 810 cm^−1^ was typical for the presence of mannose.

### 3.6. *In Vitro* Antioxidant Analysis

**Figure 5:**
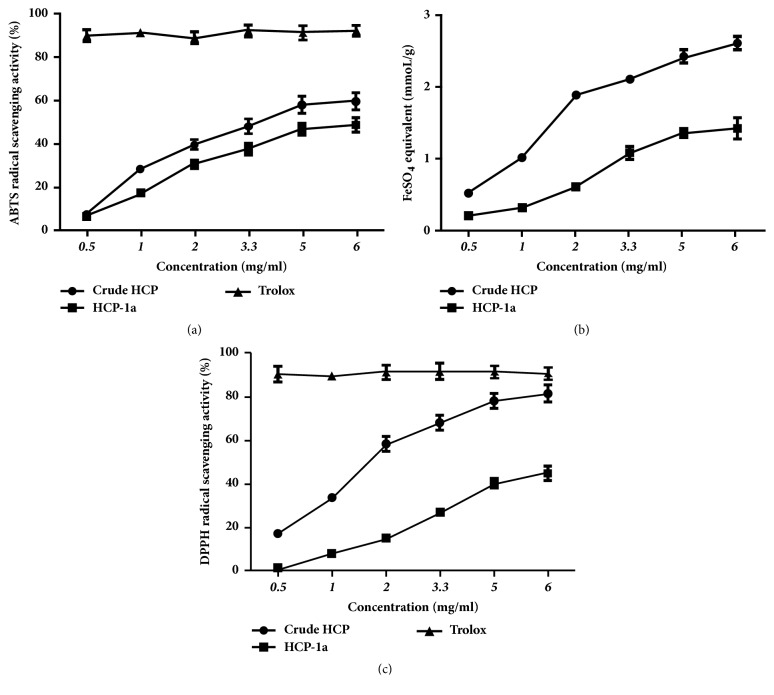
Antioxidant activity of polysaccharides. ABTS^+^ radical scavenging activity of HCP and HCP-1a (Trolox as standard control) (a); ferric reducing antioxidant power of HCP and HCP-1a (b); DPPH radicals-scavenging effect of HCP and HCP-1a (c).

The ABTS radical cation (ABTS^+^) scavenging assay is a commonly applied method to assess the antioxidant activity of most natural products [28]. Here, ABTS^+^ was employed in this study, by which the antioxidant properties of crude HCP and HCP-1a were assessed. As shown in Figure 5(a), both of the polysaccharides showed increased scavenging activities in a concentration-dependent pattern. When the tested concentration increased to 6.0 mg/mL, the scavenging abilities of crude HCP, HCP-1a, and Trolox were 60.11%, 48.88%, and 92.46%, respectively. Therefore, both of the crude HCP and HCP-1a had an appreciable ABTS^+^ scavenging activity.

The reduction of the power of Fe^3+^ to Fe^2+^ by ferric reducing antioxidant plasma (FRAP) assay is also an indicator of potential antioxidant activity of natural products [29]. As shown in Figure 5(b), the crude HCP and HCP-1a indicated different Fe^3+^ reducing activities. The FRAP values of HCP significantly increased in concentration-dependent pattern and had stronger activities than that of HCP-1a in the tested range of concentration. The highest FRAP values of crude HCP and HCP-1a were 2.61 and 1.42 mmoL FeSO_4_/g dry extract, respectively. Accordingly, crude HCP and HCP-1a may be good sources of natural antioxidants.

As a convenient method for antioxidant screening, the DPPH method is performed by forming a stable and reduced DPPH molecule through the donating of hydrogen [30].  Figure 5(c) showed the scavenging activity of crude HCP and HCP-1a on the DPPH radical compared with Trolox. Among the two polysaccharides, HCP possessed higher DPPH radical scavenging capacity compared with HCP-1a in a concentration-dependent pattern within all the tested concentration (0.5 mg/mL-6.0 mg/mL). When the concentration of HCP reached to 6.0 mg/mL, its DPPH radical scavenging capacity (82.23%) was close to Trolox (92.07%), indicating that HCP was a promising natural antioxidant.

### 3.7. Immunostimulatory Effect of Polysaccharides on Murine Macrophage Cell Line RAW264.7

**Figure 6:**
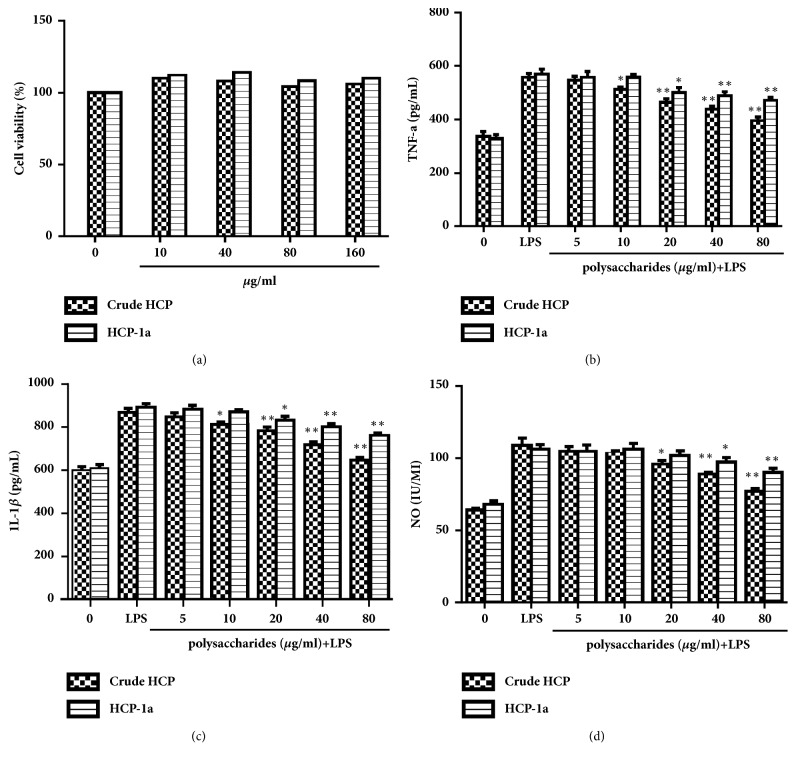
Effects of HCP and HCP-1a on cell viability, proinflammatory cytokines, and NO concentration in RAW264.7 cells. After incubating for 24 h with increasing concentrations of HCP and HCP-1a, the cytotoxicity of cells was then determined by MTT assay (a). The levels of (b) TNF-*α*, (c) IL-1*β*, and (d) NO in the collected supernatants were measured by ELISA assay. *∗∗*p<0.01; *∗*p <0.05.

The immunostimulatory activity of crude HCP and HCP-1a on murine RAW264.7 macrophage cells was investigated and commercial LPS was used for comparison. As shown in Figure 6(a), both of the crude HCP and HCP-1a did not inhibit viability of RAW264.7 macrophage cells over the concentration range tested. Therefore, these dosages were used for the treatment of crude HCP and HCP-1a in the following immunostimulatory activity experiments.

To test the immunostimulatory activity of polysaccharides, the concentrations of TNF-*α*, IL-1*β*, and NO were determined in culture supernatant when macrophages were exposed to crude HCP and HCP-1a. As shown in Figures 6(b) and 6(c), both of crude HCP and HCP-1a treatments suppressed LPS-induced TNF-*α* and IL-1*β* release in a concentration-dependent pattern while LPS treatment increased. Particularly, the production of TNF-*α* was decreased by 73.01% and 40.37% when the pretreatment of RAW was 264.7 cells by 80 *μ*g/mL of crude HCP and HCP-1a, respectively. Meanwhile, the results also indicated that crude HCP was more sensitive to TNF-*α* and IL-1*β* release than HCP-1a. As shown in Figure 6(d), LPS treatment increased NO release. But, crude HCP and HCP-1a treatment could significantly inhibit NO release in LPS-induced RAW264.7 cells. NO concentrations in RAW264.7 cells were 64.00 ± 1.34 IU/mL (68.24 ± 2.23 for HCP-1a) in a negative control and 109.03 ± 5.00 IU/mL (106.23 ± 3.24 for HCP-1a) in LPS-induced cells. NO concentrations were 77.01 ± 2.12 IU/mL (71.08% inhibition) and 90.09 ± 3.02 IU/mL (43.98% inhibition) when treated with 80 mg/mL crude HCP and HCP-1a, respectively.

NO, TNF-*α*, and IL-1*β* as important proinflammatory cytokines and inflammatory mediators, were critical in inflammation-associated and immunization-associated response [31]. Samples with NO, TNF-*α*, and IL-1*β* inhibitory activity have potential to possess immunostimulatory activity. The present study strongly suggested that the crude HCP and HCP-1a may have beneficial effects on early immunomodulation.

## 4. Conclusions

In this study, several special design methods were applied to study the extraction process of HCP, and the results were verified. The optimum conditions for enzyme-assisted extraction were determined as follows: extraction temperature 50°C, enzyme concentration 3%, and liquid-solid ratio 30ml/g. A major acidic polysaccharide fraction HCP-1a from HCP was obtained by using of column chromatography. Furthermore, compared with HCP-1, it was found that HCP had stronger antioxidant activity through evaluating in DPPH radical, hydroxyl radical scavenging, and Fe^2+^ chelating assay with comparison to Trolox. Investigation of the immunostimulatory effect of HCP and HCP-1a on RAW264.7 cells revealed that both of HCP and HCP-1a significantly upregulated the concentrations ofIL-1*β*, NO, and TNF-*α* in a dose-dependent pattern. Therefore, the present study may provide new insights into the application of polysaccharides from* Hedyotis corymbosa*. To further provide theoretical information for the application of polysaccharide from* Hedyotis corymbosa*, the detailed structure of HCP and HCP-1a and* in vivo* studies should be further investigated.

## Figures and Tables

**Table 1:** Results of ANOVA and regression analysis of CCD.

**Source**	**Sum of Squares**	**Mean Square**	**F Value**	***p*-value**	**significant**
**Model**	9.34	1.04	197.71	< 0.0001	*∗∗∗*
*** X***_***1***_	0.25	0.25	48.18	< 0.0001	*∗∗∗*
***X***_***2***_	0.031	0.031	5.99	0.0344	*∗*
*** X***_***3***_	0.34	0.34	63.80	< 0.0001	*∗∗∗*
*** X***_***1***_ *** X***_***2***_	1.42	1.42	269.92	< 0.0001	*∗∗∗*
*** X***_***1***_ *** X***_***3***_	0.49	0.49	93.95	< 0.0001	*∗∗∗*
*** X***_***2***_ ***X***_***3***_	0.050	0.050	9.47	0.0117	*∗*
***X***_***1***_^***2***^	2.68	2.68	510.57	< 0.0001	*∗∗∗*
***X***_***2***_^***2***^	4.69	4.69	892.45	< 0.0001	*∗∗∗*
*** X***_***3***_^***2***^	0.28	0.28	52.74	< 0.0001	*∗∗∗*
**Residual**	0.053	5.251E-003			
**Lack of Fit**	0.028	5.515E-003	1.11	0.4574	⧫
**Pure Error**	0.025	4.987E-003			
**Cor Total**	9.40				
**R-Squared**	0.9954				
**Adj R-Squared**	0.9894				
**Pred R-Squared**	0.9454				
**Adeq Precision**	34.815				

*∗*, *∗∗*, and *∗∗∗* represent p<0.05, p<0.01, and p<0.0001, respectively.

⧫ represents “not significant”

**Table 2:** Preliminary characterization of crude HCP and HCP-1a.

**Item**	***Crude HCP***	***HCP-1a***
**Carbohydrate (**%**)**	70.36 ± 3.86	60.95 ± 3.05
**Protein (**%**)**	1.07 ± 0.03	-
**Uronic acid (**%**)**	21.88 ± 1.06	35.26 ± 1.51
**Sulfuric radical (**%**)**	7.79 ± 0.21	1.73 ± 0.04
**Total polyphenol ** **(mg GAE /mg dried extract)**	1.89 ± 0.08	0.49 ± 0.02
**Molecular weight (KDa)**	-	33.9
**Monosaccharide composition (**%**)**		
**Man**	23.85	21.60
**Rib**	10.52	7.85
**Rha**	4.45	5.25
**GlcUA**	3.83	2.79
**GalUA:**	6.85	3.24
**Glc**	16.94	9.79
**Gal**	2.86	23.28
**Xyl**	15.50	13.88
**Ara**	15.20	9.67
**Fuc**	-	2.65

## Data Availability

The data used to support the findings of this study are available from the corresponding author upon request.

## Supplementary Materials

Supplementary Materials Supplementary Table 1: Placket-Burman design matrix and Identification of significant variables for HCP yield. Supplementary Table 2: design and results of CCD. Supplementary Table 3: experimental design and response value of path of steepest.
