# Supplementary material for: Extraction, Characterization, Antioxidant, and Immunostimulatory Activities of Polysaccharides from Hedyotis corymbosa
Source: Evid Based Complement Alternat Med. 2018 Nov 14;2018:8617070. doi: 10.1155/2018/8617070 (PMC6261247; doi:10.1155/2018/8617070)
Supplement: Supplementary Materials — Supplementary Table 1: Placket-Burman design matrix and Identification of significant variables for HCP yield. Supplementary Table 2: design and results of CCD. Supplementary Table 3: experimental design and response value of path of steepest. [file 8617070.f1.docx]

Supplementary data

**Supplementary Table 1** Placket - Burman design matrix and Identification of significant variables for HCP yield.

| Run | *X_1_*  （%） | *X_2_*  mL/g | *X_3_*  (℃) | *X_4_*  （W） | *X_5_*  （min） | *X_6_* | Yield  (‾% ) |
| --- | --- | --- | --- | --- | --- | --- | --- |
| 1 | **-1(1)** | **-1(10)** | **-1(30)** | **-1(200)** | **-1(10)** | -1(4) | 0.47 |
| 2 | 1(2) | 1(30) | -1 | -1 | 1(30) | -1 | 2.44 |
| 3 | **-1** | **1** | **-1** | **1(400)** | **1** | 1(5) | 1.07 |
| 4 | -1 | -1 | 1(60) | 1 | 1 | -1 | 1.30 |
| 5 | **-1** | **1** | **1** | **-1** | **-1** | -1 | 1.07 |
| 6 | 1 | -1 | 1 | 1 | 1 | -1 | 2.98 |
| 7 | **1** | **1** | **1** | **-1** | **1** | 1 | 3.09 |
| 8 | 1 | -1 | -1 | 1 | -1 | 1 | 1.28 |
| 9 | **-1** | **-1** | **-1** | **-1** | **1** | 1 | 0.67 |
| 10 | 1 | -1 | 1 | -1 | -1 | 1 | 1.72 |
| 11 | **1** | **1** | **-1** | **1** | **-1** | -1 | 2.54 |
| 12 | -1 | 1 | 1 | 1 | -1 | 1 | 2.01 |
| Effect | 1.2406 | 0.6347 | 0.6157 | 0.2874 | 0.4087 | -0.1584 |  |
| Coefficient | 0.6203 | 0.3173 | 0.3079 | 0.1437 | 0.2043 | -0.0792 |  |
| *t*-Value | 5.33 | 2.73 | 2.65 | 1.24 | 1.76 | -0.68 |  |
| *p*-value | 0.003* | 0.041* | 0.046* | 0.272 | 0.139 | 0.526 |  |

Press = 4.68, *R^2^* = 90.56%, *adj-R^2^* = 79.22%.

*Identified variables with a significant effect on the response (*P*<0.05).

Note: *X_1_*, Enzyme concentration; *X_2_*, Liquid-to-solid ratio; *X_3_*, Extraction temperature; *X_4_*, Ultrasonic power; *X_5_*, Extraction time; *X_6_*, PH value.

**Supplementary Table 2** Design and results of CCD.

| Run | Enzyme concentration | | liquid-to-solid ratio | | extraction time | | HCP Yield (‾%) |
| --- | --- | --- | --- | --- | --- | --- | --- |
|  | **Code *X_1_*** | ***X_1_*** (%) | **Code *X_2_*** | ***X_2_***（mL/g） | **Code *X_3_*** | ***X_3_***（℃） |  |
| 1 | 0 | 3.00 | 0 | 30.00 | 0 | 50.00 | 3.95 |
| 2 | 1 | 4.00 | -1 | 20.00 | -1 | 40.00 | 3.28 |
| 3 | -1 | 2.00 | 1 | 40.00 | -1 | 40.00 | 2.80 |
| 4 | -1 | 2.00 | -1 | 20.00 | 1 | 60.00 | 2.69 |
| 5 | -1.682 | 1.32 | 0 | 30.00 | 0 | 50.00 | 2.51 |
| 6 | -1 | 2.00 | -1 | 20.00 | -1 | 40.00 | 1.77 |
| 7 | 1 | 4.00 | 1 | 40.00 | 1 | 60.00 | 2.41 |
| 8 | 0 | 3.00 | 0 | 30.00 | 0 | 50.00 | 3.95 |
| 9 | 0 | 3.00 | 0 | 30.00 | 0 | 50.00 | 4.04 |
| 10 | -1 | 2.00 | 1 | 40.00 | 1 | 60.00 | 3.58 |
| 11 | 0 | 3.00 | 0 | 30.00 | -1.682 | 33.18 | 3.37 |
| 12 | 0 | 3.00 | 0 | 30.00 | 1.682 | 66.82 | 3.80 |
| 13 | 0 | 3.00 | 0 | 30.00 | 0 | 50.00 | 4.08 |
| 14 | 1 | 4.00 | -1 | 20.00 | 1 | 60.00 | 3.38 |
| 15 | 0 | 3.00 | -1.682 | 13.18 | 0 | 50.00 | 2.31 |
| 16 | 0 | 3.00 | 1.682 | 46.82 | 0 | 50.00 | 2.42 |
| 17 | 0 | 3.00 | 0 | 30.00 | 0 | 50.00 | 3.96 |
| 18 | 1 | 4.00 | 1 | 40.00 | -1 | 40.00 | 2.80 |
| 19 | 1.682 | 4.68 | 0 | 30.00 | 0 | 50.00 | 3.01 |
| 20 | 0 | 3.00 | 0 | 30.00 | 0 | 50.00 | 3.89 |

**Supplementary Table 3** Experimental design and response value of path of steepest ascent

| Run | Experimental value | | | Yield (‾% ) |
| --- | --- | --- | --- | --- |
|  | *X_1_(*%*)* | *X_2_*(mL/g) | *X_3_(*℃*)* |  |
| 1 | 1 | 10 | 30 | 0.52 |
| 2 | 2 | 20 | 40 | 1.63 |
| 3 | 3 | 30 | 50 | 3.90 |
| 4 | 4 | 40 | 60 | 2.49 |
| 5 | 5 | 50 | 70 | 2.86 |
| 6 | 6 | 60 | 80 | 2.71 |
| 7 | 7 | 70 | 90 | 2.53 |
